# Correlation of Comfort Score and Narcotrend Index during Procedural Sedation with Midazolam and Propofol in Children

**DOI:** 10.3390/jcm13051483

**Published:** 2024-03-04

**Authors:** Nora Bruns, Carolina A. Joist, Constantin M. Joist, Anna Daniels, Ursula Felderhoff-Müser, Christian Dohna-Schwake, Eva Tschiedel

**Affiliations:** 1 Department of Pediatrics I, University Hospital Essen, University of Duisburg-Essen, 45147 Essen, Germanyeva.tschiedel@uk-essen.de (E.T.); 2 Center for Translational Neuro- and Behavioural Sciences (C-TNBS), University Hospital Essen, University of Duisburg-Essen, 45147 Essen, Germany

**Keywords:** children, procedural sedation, propofol, midazolam, sedation monitoring, depth of sedation, Narcotrend index, Comfort scale

## Abstract

**Background/Objectives:** Precise assessment of hypnotic depth in children during procedural sedation with preserved spontaneous breathing is challenging. The Narcotrendindex (NI) offers uninterrupted information by continuous electrocortical monitoring without the need to apply a stimulus with the risk of assessment-induced arousal. This study aimed to explore the correlation between NI and the Comfort Scale (CS) during procedural sedation with midazolam and propofol and to identify an NI target range for deep sedation. **Methods:** A prospective observational study was conducted on 176 children (6 months to 17.9 years) undergoing procedural sedation with midazolam premedication and continuous propofol infusion. Statistical analyses included Pearson correlation of NI and CS values, logistic regression, and receiver operating curves. **Results:** Median NI values varied with CS and age. The correlation coefficient between CS and NI was 0.50 and slightly higher in procedure-specific subgroup analyses. The optimal NI cut-off for deep sedation was between 50 and 60 depending on the analyzed subgroup and displayed high positive predictive values for sufficient sedation throughout. **Conclusion:** Our study found a moderate correlation between NI and CS, demonstrating reliable identification of adequately sedated patients.

## 1. Introduction

Precise clinical assessment of hypnotic depth in children undergoing procedural sedation with preserved spontaneous breathing is challenging. This applies especially to the discrimination of deep from very deep or too deep sedation. At the same time, discriminating between deep, very deep, and too deep sedation is crucial to avoid complications. Several clinical sedation scales, such as the Comfort Scale (CS), Ramsay Score (RS), and the University of Michigan Sedation Scale (UMSS), are commonly used to evaluate the hypnotic state in children [1,2,3,4,5]. Their validation and performance have been assessed, with good reliability of the CS and RS [3,5,6]. Even though the CS was originally developed for sedated patients treated in the pediatric intensive care unit (PICU) and not for procedural sedation, it has been used for measuring sedation depth during procedural sedation in the PICU performed by pediatric intensivists [7,8,9,10,11]. The UMSS is a simple scoring tool and has recently shown good interrater agreement for light levels of sedation, but showed less agreement during deeper stages of sedation [12]. Even though these scores provide the possibility for objective assessment of hypnotic depth, they provide only intermittent information and can provoke assessment-induced arousal because they rely on providing a stimulus to the patient.

Contrarily, the continuous monitoring of electrocortical activity provides uninterrupted information on sedation levels without the need to apply such a stimulus. It is generally acknowledged that anesthesia-induced changes of processed EEG provide information on the hypnotic depth [13,14,15]. A long-established technique in this context is the bispectral index (BIS), which provides a dimensionless number between 0 and 100 derived from hemifrontal EEG independent of the patients’ age. An alternative technique that accounts for age-specific EEG differences is the Narcotrendindex (NI), which is processed from bifrontal EEG [16,17]. The monitor provides a categorical scale from A–F along with a number between 0 and 100.

The NI was originally developed as an EEG-based monitoring modality for general anesthesia and correlates with the minimal alveolar concentration of sevoflurane and exhaled propofol during anesthesia [18,19,20]. During transition from induction to maintenance, the use of NI can help to reduce the use of intraoperative propofol [21] and guide sedation depth during endoscopies under an opiate and propofol [22,23]. In a prospective study, patients in the NI-guided group fulfilled discharge criteria from the operating theatre earlier and spent less time outside of the target range, while there was no difference in the rating of the sedation quality [23].

The aim of this prospective observational study was to explore the correlation between the NI and Comfort scale during procedural sedation in the procedure room of a tertiary PICU. The aim was further to identify an NI target range for deep sedation for this specific sedation regime (CS < 15).

## 2. Methods

We prospectively included children between 6 months and 17.9 years of age undergoing procedural propofol sedation in the procedure room of the tertiary PICU of the University Hospital Essen between October 2020 and December 2022. Exclusion criteria were underlying neurologic diseases that impair Comfort scale scoring, known EEG abnormalities, prior participation in this study, and anticipated use of ketamine or remifentanil. Patients who unexpectedly received ketamine or remifentanil during the sedation were retrospectively excluded.

Eligible procedures were endoscopies, bronchoscopies, biopsies, and punctures including placement of drainages. Shortly after the initiation of the study, the routine sedation regimes for muscle biopsies and bronchoscopies were changed to remifentanil + propofol, causing these procedures to become ineligible from that timepoint onwards. 

### 2.1. Sedation

Sedations were performed by experienced pediatric intensivists (A.D., C.D.S., E.T.) in the procedure room of the PICU and followed international guidelines [24,25]. According to the standard sedation of our department, intravenous (i.v.) midazolam (0.05 mg/kg, maximum 2 mg) was administered as premedication, followed by an i.v. induction bolus of propofol (1 mg/kg) and continuous i.v. infusion of propofol (10 mg/kg/h). The sedation level was optimized by clinical assessment (CS target range 11–14) via administration of propofol boli (1 mg/kg) or adjustment of the continuous infusion rate as required to reach the CS target range. Sedation was immediately stopped at the end of the procedure. All patients received oxygen via a nasal cannula throughout the sedation.

### 2.2. Clinical Measurement of Sedation Depth

Sedation depth was assessed using the CS, which is the standard measure of sedation depth during procedural sedations in our department. Starting at the beginning of the sedation, the CS was recorded every five minutes and responses to intervention-related stimuli were recorded. After cessation of the procedure, a standardized painful stimulus was applied to the sternum every five minutes until eye opening after cessation of the sedation. As during t procedure, the reaction to the stimulus was recorded as part of the CS assessment.

### 2.3. Narcotrend Monitoring

For Narcotrend monitoring (MT Monitortechnik, Bad Bramsted, Germany), three hydrogel electrodes used in clinical routine care were placed on the forehead in the Fp1, Fp2, and FpZ position according to the international 10–20 system. The skin was prepared with OneStep EEG Gel Abrasiv plus^®^ (H+H Medizinprodukte, Münster, Germany) until impedance values <10 kΩ were achieved. Vital sign monitoring was performed according to the clinical routine.

### 2.4. Documentation

CS and NI were documented manually at the beginning of the sedation and every five minutes until eye opening by doctoral students uninvolved in the sedation or the performed procedure.

### 2.5. Statistical Analyses

Continuous variables are presented as mean if normally distributed and as median if skewed. Discrete variables are summarized as counts and relative frequencies. Only NI values during ongoing sedation with continuous propofol administration via a syringe pump were analyzed. 

In addition to descriptive statistics, we calculated the Pearson correlation coefficient between CS and NI. The strength of correlation was interpreted as previously suggested for medical research [26].

The discrimination of NI to predict CS values below 15 was assessed by logistic regression using a receiver operating curve (ROC) and calculating the area under the curve (AUC). The NI cut-point with the highest correct classification rate was identified using the ROCPLOT macro [27]. Sensitivity, specificity, and positive (PPV) and negative predictive values (NPV) were calculated for the identified cut-point.

Subset analyses to assess the correlation, discriminative abilities, optimal cutpoints, and sensitivity/specificity of the calculated optimal cutpoints were conducted for two subgroups of patients that received only one single procedure during the sedation: patients receiving an endoscopic intervention (including esophagogastroduodenoscopy, placement of pH-metry probe, transesophageal echocardiography, colonoscopy) and patients receiving a puncture, biopsy, or catheter placement.

All statistical analyses were performed using SAS Enterprise Guide Version 8.3 (SAS Institute Inc., Cary, NC, USA).

### 2.6. Ethics Approval

The study was approved by the local ethics committee (19-8728-BO (17 December 2019); 21-10306-BO (27 October 2021)). Written informed consent was obtained from the legal guardians of all included patients.

## 3. Results 

**Figure 1:**
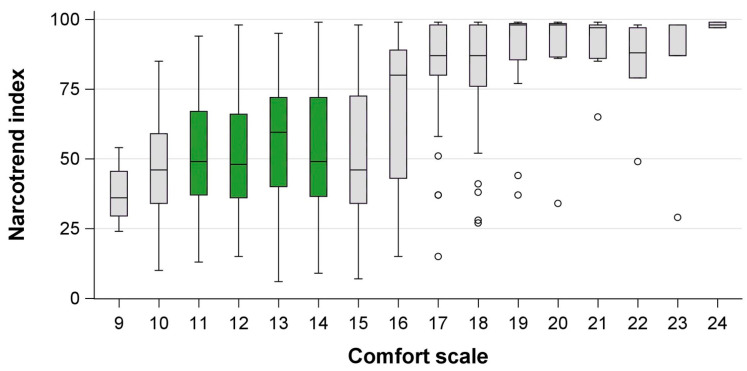
Comfort scores and corresponding Narcotrend index in children undergoing procedural sedation with midazolam and propofol. Green = Comfort scale target range, circles = outliers more than 1.5 interquartile ranges beyond the 25th/75th percentile.

**Figure 2:**
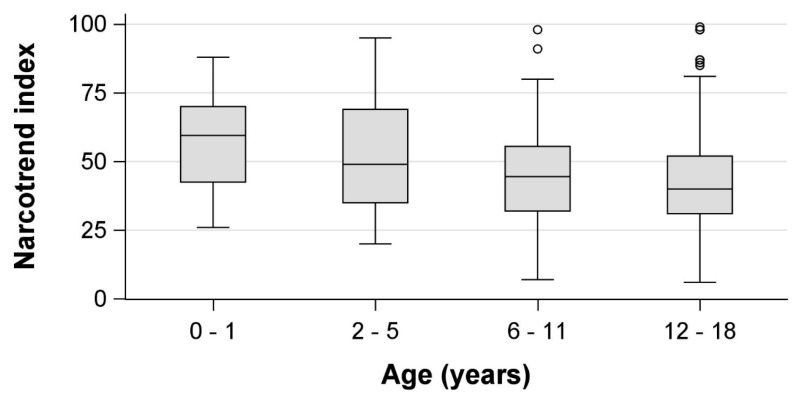
Age-dependency of Narcotrend index values in the Comfort scale target range (11–14) in children undergoing procedural sedation with midazolam and propofol. Circles = outliers more than 1.5 interquartile ranges beyond the 25th/75th percentile.

We included 188 patients, of which 12 (6%) were retrospectively excluded because ketamine or remifentanil was administered unanticipatedly during the procedure. From the remaining 176 individual patients, 867 observations of CS and NI values were obtained during ongoing sedations. Patient details are presented in Table 1 and age-specific patient details are presented in Table S1. Median NI values differed depending on the CS (Table 2, Figure 1). We observed higher median NI values in younger children, which were especially prominent in light sedation (Table 2, Figure 2). 

### 3.1. Correlation

The correlation coefficient between CS and NI was 0.50 (95% CI 0.45–0.55) for the entire cohort. For a subgroup of 37 patients receiving endoscopies, 154 paired observations of NI and CS were analyzed. The correlation coefficient was 0.62 (0.52–0.71). For 120 patients receiving biopsies/punctures/catheter placement, 533 paired observations were analyzed, showing a correlation coefficient of 0.54 (0.47–0.60).

### 3.2. Discrimination and Optimal Cut-Off Values

The AUC was 0.75 (95% CI 0.70–0.80) for the correct identification of a CS < 15 by the NI in the overall cohort. The optimal NI cut-off value to discriminate deep from non-deep sedation or wakefulness was 50. Because no difference was observed between NI values for deep sedation within the target range and too deep sedation (CS ≤ 10), a lower NI target value could not be calculated.

For the subgroup analyses of patients receiving endoscopies, the AUC was 0.79 (0.69–0.91) and the optimal cut-off to identify deep sedation (CS < 15) was 53. Analyses of patients receiving biopsies/punctures/catheter placement showed an AUC of 0.77 (0.71–0.83) and had an optimal cut-off value of 60.

### 3.3. Sensitivity, Specificity, and Predictive Values

In the complete cohort, the sensitivity and specificity of the optimum NI cut-off value were 65% (95% CI 62–69%) and 68% (61–74%), respectively, with a PPV of 88% (84–91%). The NPV was 36% (31–41%). 

For endoscopies, the sensitivity and specificity for the optimum NI cut-off value were 68% (59–76%) and 82% (69–95%), respectively. The PPV was 93% (88–98%), and the NPV was 41% (29–53%). 

In biopsies/punctures/catheter placement, the sensitivity for the optimum NI cut-off value was 61% (56–66%), the specificity was 70% (62–78%), the PPV was 86 % (82–90%), and the NPV was 37% (31–43%).

## 4. Discussion

This study assessed the utility of the NI to monitor the hypnotic depth during procedural sedation with midazolam and propofol in children. We found a moderate correlation between the clinical sedation depth and NI values and a good discrimination between light sedation and deep sedation. Median NI values in the CS target range decreased with age. The optimal NI cut-off value for the overall cohort was 50. For subgroup analyses, the optimum cut-off values differed slightly between procedures, with a higher cut-off value for punctures/biopsies/catheter placement than for endoscopies. The main and subgroup analyses showed a high positive predictive value for sufficiently deep sedation. The negative predictive value was low, indicating that sedation depth may be sufficient in spite of NI values above the cut-off. Because no difference was observed between NI values for sedation depth in the target range compared to too deep sedation, a lower target threshold could not be determined.

According to the high positive predictive value to correctly identify sufficiently deep sedation in our study, the NI may be helpful to identify patients who do not require additional sedation. Contrarily, the low NPV indicates that NI values above the optimum cut-off did not rule out sufficiently deep sedation. For that reason, NI values above 50 should not automatically prompt deepening of sedation if the patient’s clinical hypnotic depth is within the target range. The thresholds we identified in this study are lower than in two previous studies, which used 60 ± 5 and 65 ± 5 for a remifentanil plus propofol regime in endoscopies [22,23]. 

Possibly, the applied sedation regime, midazolam plus propofol, require different hypnotic depths compared to the other studies that used opiates because midazolam does not control pain. Further, substance-specific EEG changes may cause the different optimum cut-off values of the NI. For example, it has been reported that premedication with midazolam induces specific intraoperative EEG changes in elderly patients that are not observed without this premedication [28]. Another possibility is that the NI performs better in discriminating no sedation versus sedation than moderate versus deep sedation. This has similarly been reported in a pediatric study by Münte et al. that used midazolam premedication and propofol titration for anesthesia induction [29]. The NI threshold to discriminate between moderate and deep sedation was calculated at 76 by Münte et al., with sensitivity and specificity both at 74% [29]. This clearly deviates from our calculated thresholds between 50 and 60, warranting further research on the underlying reasons for the observed differences. Like Münte et al., our study found that the NI distinguished best between no sedation and light sedation and was age-dependent [29]. Generally, maturational aspects of the EEG make anesthesia and sedation monitors less reliable in young children [13,14,30]. 

As an additional consideration that might explain the deviation of the NI from previously reported target values in our study, the NI was developed to guide drug administration during anesthesia induction in the operating room to avoid over-anesthetization when switching from the induction medication to volatile anesthetics. In our patients, deeper sedation than during the induction of anesthesia may have been required because the performed procedures were invasive and potentially painful with no routinely applied intravenous analgesia. These considerations, along with our findings, call for increased attention when using the NI outside the setting it was developed for and with other sedation/anesthesia regimes than those used for development.

In our study, younger children had higher NI values while in the CS target range, pointing out the need for additional consideration when interpreting NI values. Age effects have also been reported from other forms of processed EEG during general anesthesia, such as amplitude-integrated EEG and spectral edge frequencies [31], and seem reasonable given the physiological maturation of EEG during childhood [32]. 

The main limitation of our study is the circumstance that the procedures performed in this study were heterogenous regarding duration, manipulation, and painfulness. We addressed this by performing two subgroup analyses for the procedures most frequently performed. The included patients suffered from heterogenous diseases and disease severity, but were all neurologically healthy, treated in a general pediatric ward, and the procedure was performed electively. 

Future research in the PICU setting could potentially explore the utility of the NI to measure hypnotic depth during neuromuscular blockade in critically ill children. EEG-based monitoring during neuromuscular blockade is recommended and currently assessed by BIS monitoring [33,34,35,36]. However, it has not been proven that BIS is the best measure to titrate sedation in settings where clinical sedation scales cannot be used. As the results of our study showed high positive predictive values for sufficient sedation depth, the NI may be a useful tool in this context that deserves further investigation.

In summary, this study provides evidence that the NI correlates moderately with the CS in children undergoing procedural sedation with midazolam premedication and propofol. NI values within the CS target range varied with age. An important finding was that the NI values required for sufficient clinical sedation depth were lower than in previous studies [22,23], suggesting that substance-specific effects on electrocortical activity or required sedation depth due to the used substances—or both—must be considered. According to our results, the NI reliably identifies patients who are sufficiently sedated, but is less suitable to indicate too light sedation for the applied regime with midazolam plus propofol. Our study results call for increased awareness when applying NI-based sedation depth monitoring outside of the development setting and with deviating substances.

## 5. Conclusions

This study found a moderate correlation between the NI and the CS in children receiving midazolam and propofol for procedural sedation. NI values within the CS target range varied with age. The NI reliably identified adequately sedated patients but was less reliable for correctly predicting too light sedation for the applied regime with midazolam and propofol. Our findings advise the use of additional clinical assessment tools when using NI-based sedation monitoring.

## Figures and Tables

**Table 1:** Patient characteristics, procedures, and sedation.

	N (%) *
n	176
Age [years], *mean ± SD*	9.1 ± 5.4
Age groups	
0–1 years	16 (9%)
2–5 years	49 (28%)
6–11 years	46 (26%)
12–17 years	65 (37%)
Weight [kg], *median (IQR)*	28.5 (16.8–50.0)
Procedure	
Esophagogastroduodenoscopy, placement of percutaneous gastroenterostomy, transesophageal echocardiography	46 (26%)
Colonoscopy, rectoscopy	5 (3%)
Placement of pH-metry probe	3 (2%)
Bronchoscopy	6 (3%)
Biopsy (liver, kidney, skin, muscle, thyroid)	81 (46%)
Puncture (lumbal, pleural drainage, ascites drainage, bone marrow, joint)	48 (27%)
Catheter placement or removal (central venous catheter, Shaldon, Broviac)	16 (9%)
Multiple procedures	22 (13%)
Duration of sedative administration [min], *median (IQR)*	17 (13–25)
Time until eye opening [min], *median (IQR)*	16 (10–23)
Propofol dose	
Total dose [mg/kg], *median (IQR)*	5.6 (4.0–7.6)
Induction dose via bolus application [mg/kg], *median (IQR)*	2.9 (2.0–4.1)
Maintenance dose via continuous infusion + boli [mg/kg], *median (IQR)*	2.5 (1.8–4.0)
Average infusion rate during procedure (continuous infusion + boli) [mg/kg/h], *median (IQR)*	17.7 (13.8–24.3)

* if not otherwise indicated.

**Table 2:** Narcotrend index values by Comfort scale range and age groups.

Comfort Scale	Age (Years)	n	NI Value Median (IQR)
≤10 (too deep)	Overall cohort	176	45 (33–55)
	0–1	7	42 (37–48)
	2–5	22	37 (32–51)
	6–11	18	45 (37–48)
	12–17	82	43 (28–53)
11–14 (target range)	Overall cohort	176	44 (32–63)
	0–1	40	59 (43–70)
	2–5	143	49 (35–69)
	6–11	160	44 (32–56)
	12–17	203	40 (31–52)
≥15 (not deep enough)	Overall cohort	176	81 (45–98)
	0–1	22	63 (49–97)
	2–5	50	84 (46–98)
	6–11	54	79 (40–98)
	12–17	66	98 (42–99)

IQR = interquartile range.

## Data Availability

The datasets generated will be made available to any qualified researcher upon reasonable request.

## Supplementary Materials

The following supporting information can be downloaded at: https://www.mdpi.com/article/10.3390/jcm13051483/s1, Table S1: Clinical characteristics by age groups.
