# Supplementary material for: Correlation of Comfort Score and Narcotrend Index during Procedural Sedation with Midazolam and Propofol in Children
Source: J Clin Med. 2024 Mar 4;13(5):1483. doi: 10.3390/jcm13051483 (PMC10932229; doi:10.3390/jcm13051483)
Supplement: Supplementary file 1 [file jcm-13-01483-s001.zip › jcm-2889192-supplementary.pdf]

Table S1. Clinical characteristics by age groups.

	Age group			
	**0 – 1 years**	**2 – 5 years**	**6 – 11 years**	**12 – 17 years**
n	16	49	46	65
Weight [kg], *median (IQR)*	10.2 (9.3 – 13.3)	15.0 (14.0 – 20.0)	25.9 (20.0 – 35.0)	53.0 (47.0 – 66.0)
Procedure				
Esophagogastroduodenoscopy, placement of percutaneous gastroenterostomy, transesophageal echocardiography	4 (25 %)	14 (29 %)	13 (28 %)	15 (23 %)
Colonoscopy, rectoscopy	0 (0 %)	2 (4 %)	2 (4 %)	1 (2 %)
Placement of pH-metry probe	1 (6 %)	0 (0 %)	2 (4 %)	0 (0 %)
Bronchoscopy	4 (25 %)	0 (0 %)	2 (4 %)	0 (0 %)
Biopsy (liver, kidney, skin, muscle, thyroid)	4 (25 %)	18 (37 %)	19 (41 %)	40 (62 %)
Puncture (lumbal, pleural drainage, ascites drainage, bone marrow, joint)	5 (31 %)	14 (29 %)	13 (28 %)	16 (25 %)
Catheter placement or removal (central venous catheter, Shaldon, Broviac)	1 (6 %)	5 (10 %)	4 (9 %)	6 (9 %)
More than one procedure	2 (13 %)	4 (8 %)	9 (20 %)	12 (18 %)
Duration of sedative administration [min], *median (IQR)*	24 (20 – 30)	16 (13 – 20)	17 (12 – 25)	17 (13 – 28)
Time until eye opening [min], *median (IQR)*	26.5 (21 – 33)	20 (14 – 25)	16.5 (11 – 23)	13 (8 – 17)
Propofol dose				
Total dose [mg/kg], *median (IQR)*	7.4 (6.0 – 8.9)	6.3 (6.0 – 8.9)	5.9 (4.4 – 7.0)	4.0 (3.2 – 5.4)
Induction dose via bolus application [mg/kg], *median (IQR)*	3.8 (2.8 – 4.6)	3.9 (2.8 – 4.6)	3.0 (2.4 – 4.0)	1.9 (1.3 – 2.8)
Maintenance dose via continuous infusion + boli [mg/kg], *median (IQR)*	3.9 (2.4 – 4.3)	2.4 (1.9 – 3.6)	2.6 (1.7 – 4.0)	2.4 (1.8 – 3.6)
Average infusion rate during procedure (continuous infusion + boli) [mg/kg/h], *median (IQR)*	19.0 (14.9 – 23.0)	24.5 (17.6 – 28.2)	19.3 (15.2 – 23.3)	13.9 (10.7 – 17.3)

All numbers represent n (%) if not otherwise indicated.
